# State-of-the-art surgery for pancreatic cancer

**DOI:** 10.1007/s00423-021-02362-y

**Published:** 2021-11-09

**Authors:** Anna Nießen, Thilo Hackert

**Affiliations:** Department of General, Visceral and Transplantation Surgery, University Hospital of Heidelberg, Im Neuenheimer Feld 420, 69120 Heidelberg, Germany

**Keywords:** Pancreatic cancer, Surgery, Technical advances, Extended resections

## Abstract

**Background:**

The d evelopment of surgical techniques and specialization and specifically complication management in pancreatic surgery have improved surgical outcomes as well as oncological results in pancreatic surgery in recent decades. Historical morbidity and especially mortality rates of up to 80% have decreased to below 5% today. This review summarizes the current state of the art in pancreatic cancer surgery.

**Methods:**

The present literature and clinical experience are summarized to give an overview of the present best practice in pancreatic surgery as one of the most advanced surgical disciplines today.

**Results:**

Based on the available literature, three important aspects contribute to best patient care in pancreatic surgery, namely, surgical progress, interdisciplinary complication management, and multimodal oncological treatment in case of pancreatic cancer. In addition, minimally invasive and robotic procedures are currently fields of development and specific topics of research.

**Conclusion:**

In experienced hands, pancreatic surgery—despite being one of the most challenging fields of surgery—is a safe domain today. The impact of multimodal, especially adjuvant, therapy for oncological indications is well established and evidence-based. New technologies are evolving and will be evaluated with high-evidence studies in the near future.

## Background

Historically, pancreatic cancer surgery—evolving in the second half of the twentieth century—has been burdened by a high morbidity as well as mortality rate. Following the pioneer work of Walther Kausch and Allan Whipple, who actually did operate on ampullary cancers, surgery for tumors of the head of the pancreas had been spreading around the world, initially with disappointing results [1, 2]. Because of a high percentage of surgical failure as well as disappointing oncological results, it took a long time to establish even those procedures that today are regarded as standard resections and especially to gain acceptance to perform such kind of surgery. Only during the last two decades, not only standard pancreatic resections have been established and supported by scientific evidence, but also more extended procedures have overcome the stage of experimental surgical approaches [3–6]. This has gone hand in hand with the development of modern complication management, mainly based on interventional radiological therapy—thus preventing re-operations for a large proportion of patients and improving results of surgery [7]. In addition, with regard to pancreatic cancer surgery, the implementation of adjuvant therapy has led to a substantial improvement of oncological results and justifying radical and extended surgical approaches [8, 9]. Besides this, recent developments have led to the implementation of minimally invasive and especially robotic procedures in the treatment corridors of pancreatic cancer. This reflects a general trend of extending the indications for minimally invasive as well as robotic surgery under the assumption of a potential benefit of such approaches in terms of faster recovery and—with specific regard to pancreatic cancer—allowing patients to proceed to adjuvant therapy quicker and possibly more often [10, 11]. In parallel to the above-mentioned facts, more and more evidence has been and is created on both new and advanced surgical techniques as well as on multimodal therapy strategies. This underlines the need not only to procced in a very special field of surgery but also to accompany this with studies with the aim of creating evidence and improve treatment pathways on an evidence-based level.

The aim of the present review is to give an overview on the currently available literature on technical as well as oncological developments and aspects in pancreatic cancer surgery with a critical appraisal of these topics. Furthermore, future directions and fields of surgical research are discussed.

## Surgery in a multimodal treatment approach

During the last twenty years, it has been well established that all surgical approaches to pancreatic cancer need to be supplemented by adjuvant therapy. This has been based on large multicenter randomized controlled trials such as the ESPAC or PRODIGE trials that showed a clear survival benefit when resection of pancreatic cancer was followed by adjuvant treatment with chemotherapy for 6 months [9, 12]. In contrast, no benefit for adjuvant chemoradiation could be demonstrated so far. Therefore, resection and adjuvant chemotherapy represent the standard of care today with adjuvant mFOLFIRINOX as the most effective therapy regimen, alternatively replaced by gemcitabine/capecitabine if the patient’s condition does not allow the use of mFOLFIRINOX. Other treatment protocols, i.e., gemcitabine/nab-paclitaxel could not show a superiority to gemcitabine mono which was the standard in the past and should today only be used in frail patients who cannot receive more effective therapy.

The current debate on neoadjuvant treatment is certainly the most controversial, yet evolving, field in pancreatic cancer treatment. Whereas it remains clear that resectable pancreatic cancer patients should not be treated with a neoadjuvant therapy outside clinical studies, in borderline resectable patients, the situation remains more difficult. In resectable pancreatic cancer, studies on neoadjuvant therapy have failed to show any advantage in terms of survival compared to the benchmarks set by the above-mentioned adjuvant studies achieving 2.5 to 4.5 years of median survival. In borderline resectable pancreatic cancer patients, the current evidence also fails to show a clear survival advantage, and despite of available randomized trials, their outcomes have been partly disappointing, showing median survival times of 17 months (PREOPANC) or 28 months [13]. The most recent ESPAC-5 study as well as the ALLIANCE study showed that neoadjuvant therapy by FOLFIRINOX could be effective and confirmed that the addition of radiation is probably not only ineffective but also potentially harmful [14, 15]. Yet, there is no standardization on any neoadjuvant approach and many protocols including chemotherapy or sequential chemoradiation are used around the world despite the lack of evidence.

In locally advanced pancreatic cancer, a neoadjuvant treatment is mandatory to potentially reach a state of resectability, and, comparably to the adjuvant situation, FOLFIRINOX seems to be the best regimen to choose, even in the light of lacking level 1 evidence [16].

In the context of neoadjuvant therapy, the concept of resectability is a current point of debate as well. Traditionally, resectability was based on mere anatomical criteria. Since then, there have been different concepts that respect the idea of biological tumor behavior as well as patient-related conditional factors beyond surgical aspects of technical resectability. Consequently, an anatomically resectable situation may still change to a biological or conditional borderline or even unresectable situation [17]. These considerations underline the ongoing development of selecting the right patients who benefit from surgery—an unquestionable purpose that neoadjuvant treatment may serve for and which is still undergoing considerable changes with new criteria being proposed, including inflammatory response, liquid biopsy markers, and genomic mutations [18].

## Technical aspects of pancreatic cancer surgery

### General surgical techniques

Several substantial modifications have been introduced since Allan Whipple promoted partial pancreatoduodenectomy which had been originally described by Walther Kausch more than a century ago [19]. The initial two-staged procedure has been modified by Whipple himself to a one-stage operation, anecdotally caused by the visit of a group of European surgeons coming to Columbia University in New York to learn about the new procedure and stimulating Whipple to perform the operation in one step. After the classical Whipple operation had been performed for four decades with a resection of the stomach, Traverso and Longmire introduced the modification of a pylorus-preserving procedure without compromising oncological radicality [20]. Though being regarded as the more physiological procedure, pylorus preservation has been suspected to be a promoting factor for the complication of delayed gastric emptying (DGE)—a still poorly understood and unsolved clinical problem. However, recent randomized controlled trials on this topic have investigated the resection of the pylorus to reduce DGE without confirming a beneficial effect of pyloric resection [21]. Consequently, today’s standard of care remains the pylorus preserving modification. The same observation with regard to DGE has been made for the route of reconstruction, namely, the question if an ante- or retrocolic route may be superior in order to reduce DGE. Meanwhile, high-level evidence has shown that both options are possible and do not significantly impact the incidence of DGE, leaving the decision up to the surgeon’s discretion [22].

Other specific aspects in pancreatic cancer surgery include the techniques of “artery first,” “uncinate first,” “triangle resection,” “arterial divestment,” “RAMPS,” “DP-CAR,” and venous as well as arterial vascular resections. “Artery first” described the technique of approaching the superior mesenteric artery as the initial step of the procedure to evaluate a possible arterial tumor involvement. The mesenteric artery can be approached from different directions with at least six pathways having been described [23]. These include left or right infracolic as well as superior transmesocolic approaches. These approaches should be routinely used especially in case of any doubt of an arterial involvement. A recent meta-analysis comparing artery-first vs. standard resection with more than 1400 patients showed less blood loss and transfusion as well as a higher rate of R0 resections and a shorter hospital stay when an artery-first approach was used [24]. In contrast, these findings were not confirmed by a randomized trial including 153 patients that failed to confirm the advantage in terms of a radical R0 resection [25]. However, it has to be kept in mind that the initial step of approaching the artery first does not automatically imply a true radical resection technique afterwards as the number of harvested lymph nodes was similar in this study, but there was a considerably higher rate of R1 resections in positions other than the medial margin (i.e., pancreatic transection margin 7% standard vs. 28% artery first). This underlines that an artery first approach needs to be combined with a radical soft tissue removal (see below). The “uncinate first” approach describes a technique where the resection is carried out in a caudo-cranial technique, thereby starting with the dissection of the uncinate process from the transverse mesocolon and especially the superior mesenteric vein and artery—in contrast to the classical dissection starting with the division of the cranial portion of the retropancreatic soft tissue [26]. The uncinate first technique allows a meticulous control of the vessels from the beginning of the resection phase on and requires a division of the first jejunal loop followed by skeletonization and transposition of the loop to the right side of the mesenteric axis in the beginning of the resection phase. After lifting the specimen on the right side, the mesenteric vessels are clearly exposed and guide the further resection until the skeletonization is completed. It can be combined with an artery first approach and other techniques and be performed laparoscopically or robotically. Regarding procedure-specific outcomes, lymph node harvest, R0 resection rates, operative time, and median length of hospital stay at least equal conventional approaches, some studies even report superior outcomes with less blood loss and a shorter time to oral intake [27, 28].

With the increasing application of neoadjuvant therapy in pancreatic cancer, especially in locally advanced disease, surgical strategies and concepts have been developed for cases which have been downstaged or shown a stable course. The “triangle” operation was initially described as a method of radical resection after neoadjuvant therapy in locally advanced pancreatic cancer [29]. The rationale of the procedure is the observation that after neoadjuvant therapy, conventional imaging fails to differentiate between actual tumor abutment/encasement and fibrotic residual tissue around the vascular structures. The technique comprises a frozen section proof of no viable tumor tissue around the arterial structures (celiac axis, hepatic artery, superior mesenteric artery) allowing a sharp dissection without an arterial resection or reconstruction with the removal of all lymphatic and neural tissue structures to the basis of celiac axis and superior mesenteric artery. The venous porto-mesenteric vessels—in contrast to the arterial structures—have to be resected and replaced during the procedure in a high proportion of patients. This radical artery-sparing approach results in an anatomic triangle bordered by the superior mesenteric artery, celiac axis, and portal vein. It is essential that the arteries are reached on the adventitial layer which opens longitudinally and allows for complete lymphadenectomy and soft tissue removal of the respective area. Besides its use in locally advanced pancreatic cancer, a triangle operation can—and potentially should be—carried out in all resectable as well as borderline resectable tumors in the light of a truly radical surgical approach [30, 31].

Another related technical approach in patients after neoadjuvant therapy has been described as the “periarterial divestment” technique [32]. This technique comparably aims at a radical tumor clearance without arterial resection and is characterized by entering the adventitial layer between the arterial wall and remnant tumor/fibrotic tissue. Once this layer is opened, it is the guiding plane for dissection and allows the surgeon to avoid an arterial resection in a considerable proportion of patients.

Radical antegrade modular pancreatosplenectomy (RAMPS) describes the systematic radical surgical approach in distal pancreatectomy [33]. It is characterized by anatomic landmarks including the left-sided portal vein as well as the aorta with the celiac axis and superior mesenteric artery as the left-sided borders and the left kidney vein and diaphragm as the inferior and superior borders, respectively. Posteriorly, the location and extension of the tumor defines the choice of procedure. This is either an anterior RAMPS including Gerota’s fascia, the prerenal fat on the surface of the adrenal gland and upper half of the kidney or a posterior RAMPS which includes the left adrenal gland, all retroperitoneal fat tissue and leaving the muscle layer of the abdominal wall as the posterior border of dissection. This procedure can lead to R0 resection rates as high as 90%’ however, its influence on recurrence-free and overall survival remains controversial.

Distal pancreatectomy with celiac axis resection (DP-CAR) is a modification of the Appleby procedure, originally developed for gastric cancer surgery [34]. It is suitable for a subgroup of pancreatic cancer patients in whom only the celiac axis is involved, whereas aorta, superior mesenteric artery, and gastroduodenal artery are not involved. In this patient group, DP-CAR allows a radical tumor removal and can lead to a margin-negative resection with a median overall survival ranging between 16 and 32 months comparable to resectable pancreatic cancer [35]. The crucial point in this procedure is the arterial liver perfusion via retrograde inflow from the superior mesenteric artery, inferior pancreatico-duodenal artery, and the gastroduodenal artery. Some centers have described the technique of preoperative hepatic artery embolization to precondition arterial flow and finally allow DP-CAR in a larger proportion of patients than without embolization [36]. Despite such modifications, DP-CAR has traditionally been burdened by high rates of morbidity (50–80%) as well as mortality, varying between 3.5 and 17% [35]. As shown for many surgical pancreas procedures, these outcomes are depending on the center’s case load which result in three- to five-fold differences in mortality (18% in low volume vs. 3.5–6% in high volume centers, respectively). Furthermore, oncological outcomes of DP-CAR can be improved when embedded in a neoadjuvant therapy concept.

Overall, artery first, uncinate first, triangle operation, periarterial divestment, RAMPS, and DP-CAR are complementary modern techniques in pancreatic cancer surgery which are vessel-oriented and allow not only resections in patients that historically were regarded as unresectable, but also the removal of all putatively tumor-infiltrated soft tissue with the aim of an increased rate of R0 resection combined with a decreased risk of local recurrence.

## Vascular resection techniques

### Venous resection

Vascular resection during pancreatic cancer surgery has been described since the 1950s [37]. However, until the 1990s, there has not been a widespread acceptance for such procedures, despite reports describing en bloc pancreatoduodenectomy with vein resection with reasonable results [38]. In the meantime, venous resections have become a routine surgical procedure in high volume centers and have been applied for all types of resections including pancreaticoduodenectomy, distal, or total pancreatectomies. Depending on tumor location and extent of resection, four types of venous reconstruction can be differentiated and have been defined [39]. Surgical outcomes show that pancreatectomy with venous resection requires longer operative time and leads to an increased blood loss compared to standard resections although overall postoperative morbidity is comparable [40]. As venous resections are performed for more advanced tumors, they may be burdened by lower R0 rates and more positive lymph nodes. Based on these findings, from the oncological point of view, patients with venous resection may show lower 1-year, 3-year, and 5-year overall survival rates [41]. Yet, this remains controversial as a recent propensity score-matched analysis showed similar survival in pancreaticoduodenectomy with venous resection compare to standard pancreaticoduodenectomy after adjustment for baseline characteristics. Also, a Japanese study demonstrated a 30-month median survival time in these borderline resectable patients with venous resection in combination with adjuvant therapy, which is comparable to the survival of patients without venous resections [42].

This underlines the need to perform a venous resection whenever required to achieve negative resection margins and not to compromise radicality by avoidance of vascular resection. However, the effects of neoadjuvant therapy in this setting cannot be finally answered until results from larger high-level evidence studies are available.

### Arterial resection

In contrast to DP-CAR as an arterial resection where no vascular reconstruction is required, pancreatic resections with major arterial reconstruction have had—and partially still have—a questionable reputation due to both a high morbidity and mortality as well as potentially poor oncological outcomes [43]. However, in recent years, several large studies have demonstrated that these problems can be overcome by passing a learning curve with a respective case load and complication management facilities as well as a multimodal treatment approach. Single-center series including between 34 and 195 patients show good surgical outcomes with morbidity and mortality rates of approximately 50% and mortality of as low as 3–5% in experienced hands [44–46]. Furthermore, median survival times of 29 months and up to 25% 5-year survival justify this approach as an important tool in modern pancreatic cancer surgery after proper patient selection.

### Minimally invasive and robotic surgery

Open surgery remains the standard of surgical care for partial and total pancreatoduodenectomy. Some recent studies show that minimally invasive techniques (laparoscopic/robotic) can reduce the morbidity of pancreatectomies without having a negative impact on cancer outcome. After two randomized controlled trials showed potential advantages of laparoscopic pancreatoduodenectomy, these results were challenged by the Dutch LEOPARD 2 trial which reported an increased mortality in the laparoscopic group and was—therefore—stopped prematurely [47]. A subsequent meta-analysis, however, did not confirm the potential inferiority of the laparoscopic approach, but rather showed that applying laparoscopic techniques is a matter of learning curve and case load [48]. In line with this, a recent Chinese multicenter randomized trial on laparoscopic vs. open partial pancreatoduodenectomy including 656 patients has shown similar perioperative outcomes in terms of morbidity, mortality, and radicality of resection [49]. In the laparoscopic group, hospital stay was reduced by one day as the only significant result. Thus, as this difference is marginal, long-term results are not available, and this procedure is associated with a long learning curve; it cannot be recommended as a routine approach.

In contrast, minimally invasive distal pancreatectomy can be recommended as the standard approach for tumors of the body and the tail of the pancreas. There is level I evidence for a superiority in terms of postoperative delayed gastric emptying, time to functional recovery, and length of hospital stay [50, 51].

Robotic approaches have to date only been reported in observational patient cohorts. Therefore, no evidence-based recommendation can currently be given. In centers with the respective expertise, robotic approaches seem to be not only feasible but also potentially advantageous in terms of blood loss, time to recovery, and length of hospital stay without compromising oncological radicality [52]. Also, extended robotic approaches in pancreatic cancer surgery, including pancreatoduodenectomy with venous resection and DP-CAR, have been reported with good outcomes. These findings remain to be confirmed in larger series and are—as all advanced surgical procedures—depending on an extensive experience of the surgeon as well as the center. Current randomized and thereby high-level evidence studies on the impact of robotics in pancreatic cancer surgery are ongoing and focus not only on perioperative but also on long-term oncological results.

## Complication management

The importance of an adequate complication management in pancreatic surgery is unquestionable, and the contribution of an interdisciplinary team approach to this—including interventional radiologists, anesthesiologists, endoscopists, and surgeons—is an essential backbone of the surgical treatment of pancreatic cancer patients. As soon as 2003, it was shown that radiological interventions, mainly for intraabdominal fluid collections due to undrained postoperative pancreatic fistulas but also for control of arrosional postpancreatectomy hemorrhage, can prevent the need for re-operation in a high proportion of patients postoperatively and reduces morbidity-associated mortality [7]. A further field of non-surgical complication management includes the interventional therapy for postoperative bile leakage following pancreatoduodenectomy by percutaneous transhepatic cholangio-drainage as well as endoscopic therapy of intraluminal bleeding (i.e., located at the duodeno- or gastro-jejunostomy) and endoscopic ultrasound-guided drainage of postoperative fluid collections following distal pancreatectomy. All of these measures certainly need to be accompanied by an appropriate intensive care medicine therapy if necessary. If this is not guaranteed, complications need to be solved surgically which is not state of the art today as it is burdened by a very high subsequent morbidity and mortality which has been well investigated and shown to be inferior—even finally resulting in a failure to rescue. Therefore, surgical complication management is reserved for early (< 24 h) postoperative complications such as immediate postoperative bleeding which can easily be managed by re-operation. In all other situations, a surgical re-intervention, such as for complicated septic postoperative fistula or uncontrolled late postpancreatectomy hemorrhage, must be regarded as an “ultima ratio” approach as outcome is often poor. Despite this restrictive policy, there are certain patients who do require surgical management. A recently described and still controversially discussed situation for a (potentially early) re-operation with remnant pancreatectomy is the occurrence of a severe postoperative remnant pancreatitis, an event occurring in up to 15% of all patients after partial pancreatoduodenectomy. If not managed surgically, this complication leads to a fatal outcome in up to 55% of the patients [53, 54].

## Future directions

The future of pancreatic cancer surgery comprises three main aspects. The first aspect is the upcoming stratification of patients who will benefit from a resection and the definition of the correct pathways regarding upfront resection and adjuvant therapy or the choice of neoadjuvant therapy. If neoadjuvant therapy is chosen, the correct type of therapy needs to be defined, potentially not based on anatomical criteria or other routine markers available today, but rather on molecular stratification retrieved by tissue or liquid biopsy. The second aspect is the impact of minimally-invasive and robotic procedures that will gain importance and – comparably to the above mentioned – the challenge will be the correct selection of patients for such procedures. With the implementation of image guidance and other upcoming technologies, these procedures will be extended and potentially allow very precise and eventually also individually tailored resections. Thirdly, as a general conclusion of the latter points, pancreatic cancer treatment—either by surgery or by medical oncological medical approaches alone—will be a subject of individualized and patient targeted considerations as this can be expected for most fields of oncology in the future. These decisions could be supported by artificial intelligence and its possibility to create therapy algorithms that are not yet possible to date. Still, there is an urgent need to proceed with research in this field to allow for best treatment options and therapy decisions as pancreatic cancer is still on the rise and is projected to represent the second most common cause of cancer death in the Western countries in the next decade.

## Conclusion

During the past two decades, surgery for pancreatic cancer has been changing and developing rapidly with specific technical approaches. These techniques and an adequate perioperative complication management allow extended resections with low mortality and strengthen the impact of surgery as the backbone of any potentially curative approach for pancreatic cancer patients. In addition, multimodal treatment has evolved mainly with the establishment of adjuvant chemotherapy as the standard of care for every patient. The impact of neoadjuvant treatment is unquestionable in patients diagnosed with locally advanced pancreatic cancer and allows resection in a considerable proportion of these patients afterwards. Neoadjuvant therapy for resectable cancer is not based on any high-level evidence; however, in borderline resectable situations, studies are ongoing and will provide data in the near future. New technical approaches including robotic resections are emerging, and their actual value is intensely investigated.
